# Enhanced Recovery After Surgery Incorporating Erector Spinae Plane Block Versus Standard Care in Adolescent Idiopathic Scoliosis: A Comparative Cohort Analysis of Early Postoperative Recovery

**DOI:** 10.3390/medicina62040775

**Published:** 2026-04-16

**Authors:** Sergio De Salvatore, Gianmichele Di Cosimo, Paolo Brigato, Michele Inverso, Leonardo Oggiano, Sergio Sessa, Davide Palombi, Francesca Palmieri, Stefano Guida, Antonio Contursi, Caterina Fumo, Cloe Curri, Sebastian Miccio, Maria D’Alessandro, Pier Francesco Costici

**Affiliations:** 1 Orthopedic and Traumatology Unit, Department of Surgery, Bambino Gesù Children’s Hospital, 00165 Rome, Italy; 2 Department of Anesthesia and Critical Care, IRCCS, Bambino Gesù Children’s Hospital, 00165 Rome, Italy; 3 Research Unit of Orthopaedic and Trauma Surgery, Department of Medicine and Surgery, Università Campus Bio-Medico di Roma, Via Alvaro del Portillo, 00128 Rome, Italy; paolo.brigato@outlook.it (P.B.); davidepalombi@hotmail.it (D.P.); 4 Management and Diagnostic Innovations & Clinical Pathways Research Area, Neurorehabilitation and Adapted Physical Activity Day Hospital, Bambino Gesù Children’s Hospital, IRCCS, 00165 Rome, Italy

**Keywords:** adolescent idiopathic scoliosis, enhanced recovery after surgery, ERAS, AIS, erector spinae plane block, posterior spinal fusion, pain, nausea, length of stay, pediatric spine surgery

## Abstract

*Background and Objectives*: Enhanced Recovery After Surgery (ERAS) pathways are increasingly used in spine surgery, but uptake in adolescent idiopathic scoliosis (AIS) remains heterogeneous across institutions. Evidence in pediatric deformity surgery supports shorter recovery with protocolized care, yet real-world comparative data combining ERAS and the erector spinae plane block (ESPB) remain limited. This study aimed to compare early postoperative outcomes between a historical standard-care pathway and a structured ERAS+ESPB pathway in adolescents undergoing posterior spinal fusion for AIS. *Materials and Methods*: A single-center retrospective time-based comparative cohort study design included consecutive AIS patients (<18 years) treated between 1 January 2024 and 31 December 2025. The standard-care pathway was applied to patients operated on before 1 June 2025 (n = 34), whereas the ERAS+ESPB pathway was applied to those operated on from 1 June 2025 onward (n = 35), following formal institutional implementation. Outcomes included postoperative pain assessed using the visual analog scale under two functional conditions—at rest in the supine position and during standing/mobilization—at POD0, POD1, POD2, POD3, discharge, and 2-week follow-up; postoperative nausea at POD0–POD3; and length of stay (LOS). Between-group pain comparisons used Welch’s t-test; nausea used Fisher’s exact test; LOS used the Wilcoxon rank-sum test. *Results*: At POD0, supine pain was lower in ERAS+ESPB (1.50 ± 0.55) than in standard care (3.20 ± 1.50; *p* < 0.001). From POD1 onward, supine pain did not differ significantly between groups. Among assessable patients, standing pain was lower in ERAS+ESPB at POD2 (3.05 ± 1.53 vs. 4.50 ± 1.05; *p* = 0.020), POD3 (2.82 ± 1.62 vs. 4.17 ± 1.03; *p* = 0.006), and 2-week follow-up (1.45 ± 0.80 vs. 2.26 ± 0.93; *p* = 0.006). Nausea was lower in ERAS+ESPB at POD0 (11.4% vs. 35.3%; *p* = 0.024) and POD2 (8.6% vs. 32.4%; *p* = 0.018), with no significant differences at POD1 or POD3. LOS was shorter in ERAS+ESPB (5.41 ± 1.10 vs. 8.32 ± 2.06 nights; *p* < 0.001). *Conclusions*: In adolescents undergoing posterior spinal fusion for AIS, an ERAS-based perioperative pathway incorporating ESPB was associated with improved early postoperative recovery, particularly in terms of immediate postoperative pain, pain during mobilization, early postoperative nausea at selected time points, and length of hospital stay. Prospective multicenter studies are needed to confirm these findings and clarify the independent contribution of individual pathway components.

## 1. Introduction

Adolescent idiopathic scoliosis (AIS) is the most common spinal deformity in otherwise healthy adolescents and remains a major indication for posterior spinal fusion when curve progression, deformity severity, or functional impact justify surgical correction [1,2]. Despite advances in instrumentation and perioperative care, AIS surgery is still associated with a substantial early postoperative burden, particularly in terms of pain, opioid exposure, nausea, delayed mobilization, and prolonged inpatient recovery [3]. These outcomes are particularly relevant in pediatric populations, where rapid functional recovery and reduction in treatment-related distress are central goals.

Enhanced Recovery After Surgery (ERAS) has progressively transformed perioperative medicine through standardized, multidisciplinary pathways designed to attenuate surgical stress, improve recovery quality, and shorten hospital stay [4]. In adult spine surgery, ERAS principles have already been formalized in consensus recommendations, with a broad emphasis on multimodal analgesia, early feeding, early mobilization, and minimizing the use of tubes and drains [5]. In this context, ERAS is no longer considered an experimental framework in adults but rather an increasingly consolidated strategy for perioperative optimization.

By contrast, translation of ERAS principles to AIS populations has been slower and more heterogeneous across institutions. Early pediatric spine “rapid recovery” pathways demonstrated that protocolized multimodal care can improve pain trajectories and accelerate key recovery milestones after posterior fusion [6]. Subsequent evidence syntheses in AIS have reinforced this signal: prior meta-analytic data reported a consistent reduction in length of stay without a clear increase in complications or readmissions [7]. More recently, the updated systematic review and meta-analysis by Brigato et al. further supported the effectiveness and safety profile of ERAS-oriented approaches in AIS, while also highlighting persistent inter-study variability in protocol composition and implementation intensity [8]. The current literature supports ERAS in pediatric deformity surgery, but also indicates that center-specific pathway design can meaningfully influence outcomes.

Despite this growing body of evidence, the implementation of ERAS pathways in AIS remains heterogeneous, with considerable variability in protocol composition, timing of adoption, and integration of regional anesthesia techniques. As a result, real-world data reflecting routine clinical implementation are essential to understand how pathway-based strategies perform outside controlled or highly protocolized environments.

Within multimodal perioperative analgesia, the erector spinae plane (ESP) block has emerged as a promising adjunct in AIS surgery. Contemporary pooled evidence suggests that ESP-based strategies may improve early postoperative analgesia and contribute to opioid-sparing effects, with potential downstream benefits on nausea and mobilization metrics [9]. However, real-world comparative datasets integrating a full ERAS pathway plus an ESP block versus traditional care remain limited, particularly in single-centre pediatric spine practice, where implementation is often stepwise and historically constrained by local routines.

There is limited evidence evaluating ERAS pathways that explicitly incorporate ESPB within a comprehensive perioperative framework, as opposed to isolated analgesic interventions. This distinction is clinically relevant because ERAS is inherently a bundled strategy, and outcomes may depend on the interaction between its individual components.

The present study was designed to address this gap by comparing an ERAS pathway incorporating ESP block with a conventional postoperative protocol in AIS patients undergoing posterior spinal fusion. The primary objective was to evaluate early postoperative recovery through clinically meaningful endpoints: pain (supine and standing), postoperative nausea, and length of hospital stay. By focusing on these outcomes, this analysis aims to provide practice-oriented evidence on whether protocol modernization translates into measurable short-term benefits in adolescent spine surgery.

## 2. Methods

### 2.1. Study Design and Setting

This was a single-centre, time-based comparative cohort study conducted at Bambino Gesù Children’s Hospital in Rome. The study evaluated early postoperative recovery outcomes in adolescents undergoing elective posterior instrumented fusion for AIS, comparing a historical standard-care pathway with a subsequently implemented ERAS pathway that included an erector spinae plane block (ESPB).

### 2.2. Study Period and Patient Selection

All consecutive eligible patients who underwent surgery between 1 January 2024 and 31 December 2025 were screened.

#### 2.2.1. Inclusion Criteria

Patients were included if they met all of the following:

1. Diagnosis of adolescent idiopathic scoliosis (AIS);
2. Age < 18 years at the time of surgery;
3. Elective primary posterior instrumented spinal fusion performed at the study center;
4. Availability of postoperative data for the prespecified early outcomes (pain, nausea, and length of stay).

#### 2.2.2. Exclusion Criteria

Patients were excluded if any of the following applied:

1. Age ≥ 18 years;
2. Non-idiopathic scoliosis (congenital, neuromuscular, syndromic, or other secondary deformities);
3. Revision spinal surgery or previous major spinal procedure;
4. Non-comparable perioperative pathway;
5. Missing or incomplete data for primary postoperative endpoints.

### 2.3. Cohort Allocation

Cohort assignment was determined by the date of surgery, reflecting institutional clinical practice:

Standard-care cohort: surgeries performed before 1 June 2025;

ERAS+ESPB cohort: surgeries performed from 1 June 2025 onward, following formal ERAS implementation.

This represented a pragmatic before–after comparison within the same center.

### 2.4. Perioperative Pathways

#### 2.4.1. Historical Standard-Care Pathway

During the first 48 postoperative hours, patients received morphine-based analgesia (0.1 mg/kg over 24 h). This was followed by a 24-h phase with ketorolac (Toradol) plus tramadol, after which analgesia was maintained with scheduled ketorolac and paracetamol for the remainder of hospitalization. In addition, a nasogastric tube was routinely used in the immediate postoperative period and maintained for the first 24 h.

#### 2.4.2. ERAS+ESPB Pathway

**Figure 1:**
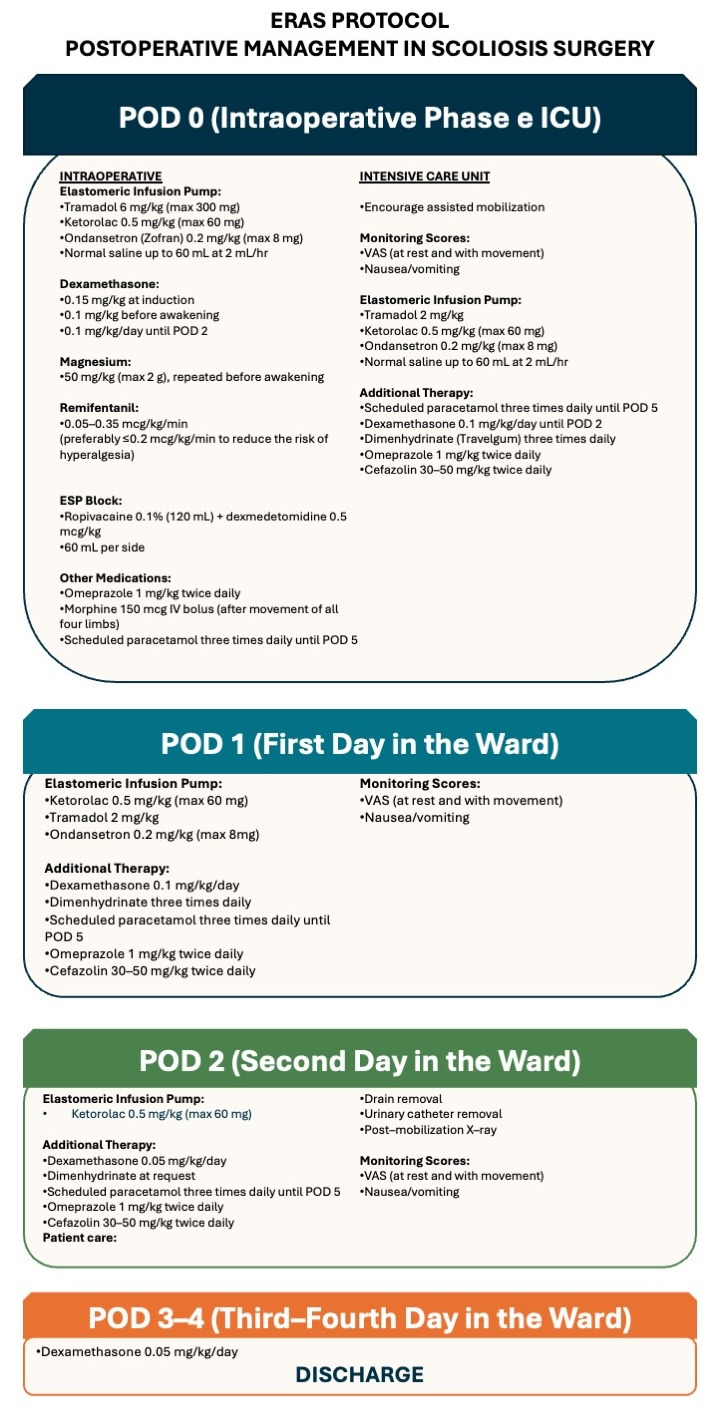
ERAS Protocol.

From 1 June 2025 onward, patients were managed with a structured multidisciplinary ERAS pathway (spine surgery, anesthesia, nursing, physiotherapy), based on multimodal opioid-sparing analgesia, standardized antiemetic management, and accelerated recovery milestones. A schematic overview of the ERAS protocol is provided in Figure 1.

As part of the antiemetic and gastrointestinal recovery strategy, Travelgum (dimenhydrinate chewing gum) was introduced postoperatively according to institutional practice, with the aim of reducing nausea and promoting bowel reactivation through chewing.

In both groups (standard technique and ERAS), patients were admitted to the Intensive Care Unit (ICU) for the first 24 h, as per standard hospital protocol.

Both perioperative pathways reflected standardized institutional protocols in use during the respective time periods and were consistently applied to all eligible patients. No case-by-case customization outside protocol indications was performed, ensuring internal consistency of pathway implementation.

### 2.5. ESPB Technique

In the ERAS cohort, the ESP-plane infiltration was performed under direct surgical exposure at wound closure, infiltrating paravertebral tissues and the dorsolumbar fascia bilaterally.

The injectate was standardized as follows:

Ropivacaine (AstraZeneca, Cambridge, UK) 0.1% (Naropin): total volume 120 mL (total dose 120 mg);
Dexmedetomidine: total dose 60 mcg.
The solution was equally divided between the sides: 60 mL per side (i.e., 60 mg ropivacaine + 30 mcg dexmedetomidine per side).
Preparation was performed using six 20 mL syringes; each contained 20 mg ropivacaine and 10 mcg dexmedetomidine.

### 2.6. Mobilization Policy

In both cohorts, first assisted mobilization was routinely attempted at approximately 24 h postoperatively. If clinically relevant nausea or lipotymic symptoms occurred, the mobilization attempt was interrupted and recorded as not tolerated at that timepoint.

### 2.7. Outcomes

The study focused on three early postoperative outcomes:

1. Postoperative pain (visual analog scale), assessed at POD0, POD1, POD2, POD3, discharge, and 2-week follow-up, under two conditions:

Supine pain;
Standing pain.

2. Postoperative nausea, recorded daily (yes/no) on POD0–POD3.
3. Length of stay (LOS), defined as the number of nights from surgery to hospital discharge.

At each predefined postoperative timepoint, assisted mobilization was attempted in both cohorts according to routine clinical practice. Standing pain was recorded only when the patient was able to reach and tolerate the upright position sufficiently to provide a valid pain score. If mobilization was attempted but could not be completed because of pain, opioid-related symptoms, nausea, lipotymic symptoms, or poor tolerance, standing pain at that timepoint was classified as not assessable and was not imputed. Pain was assessed separately in the supine and standing positions to distinguish resting pain from dynamic pain during mobilization. This distinction is clinically relevant because early functional recovery, including tolerance of upright posture and assisted ambulation, is a central component of ERAS pathways.

### 2.8. Patient Characteristics, Intraoperative Variables, and Data Collection

Patient characteristics collected from institutional electronic records included age, sex, preoperative major curve magnitude (main Cobb angle), and curve pattern according to Lenke classification. In addition, intraoperative variables included operative time and estimated blood loss. All data were extracted using standardized abstraction procedures and anonymized before analysis. Preoperative and intraoperative between-group characteristics are reported in Table 1.

Data extraction was performed retrospectively by trained investigators using institutional electronic records, with predefined variable definitions to ensure consistency and reproducibility of data collection.

### 2.9. Statistical Analysis and Ethics

Continuous variables are presented as mean ± standard deviation (SD), and categorical variables as counts and percentages. All tests were two-sided, with statistical significance set at *p* < 0.05.

Between-group pain comparisons at each timepoint were performed using Welch’s two-sample *t*-test.

LOS was compared using the Wilcoxon rank-sum test.

Postoperative nausea was analyzed separately at each postoperative day (POD0–POD3) using two-sided Fisher’s exact test. Given the exploratory nature of the study and the limited sample size, these analyses were interpreted descriptively.

Because of the non-randomized before–after design, an exploratory multivariable linear regression analysis was performed for length of stay as a complete-case analysis. The model included treatment group, age, sex, main Cobb angle, estimated blood loss, and operative time as prespecified covariates. Given the limited sample size and the retrospective nature of the dataset, the adjusted analysis was restricted to the primary recovery outcome and interpreted cautiously.

Given the exploratory nature of these analyses, *p*-values were interpreted descriptively; no multiplicity adjustment was applied. No imputation was performed; analyses were conducted on available data for each endpoint/timepoint. Statistical analyses were performed in R Studio (version 4.3.2, R Foundation for Statistical Computing, Vienna, Austria).

This study was conducted in accordance with international ethical standards for clinical research as outlined in the Helsinki and Istanbul Declarations. Formal ethical approval was not required, in accordance with institutional policy and applicable national and international regulations, because the study was retrospective and based exclusively on fully anonymized data collected during routine clinical practice.

## 3. Results

### 3.1. Study Population

A total of 69 adolescents with AIS were included in the final analysis: 34 in the standard-care group and 35 in the ERAS group.

### 3.2. Postoperative Pain

#### 3.2.1. Supine Pain

Supine pain was evaluated from POD0 to 2-week follow-up. At POD0, the ERAS group had lower supine pain than the standard care group (1.50 ± 0.55 vs. 3.20 ± 1.50). Detailed supine pain values at each timepoint are reported in Table 2.

**Figure 2:**
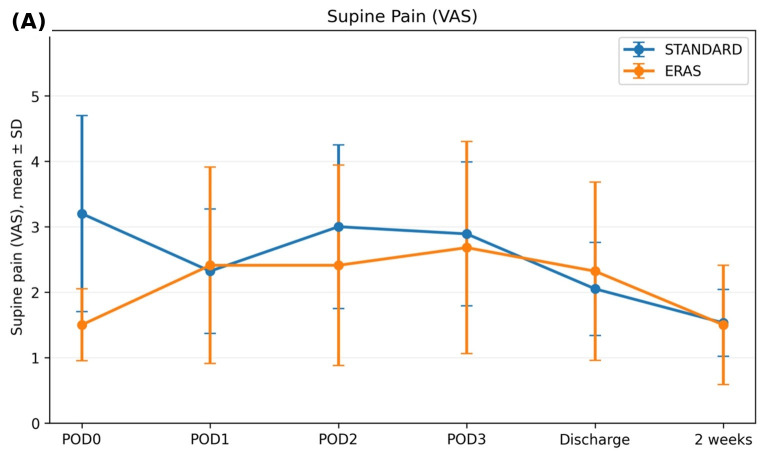

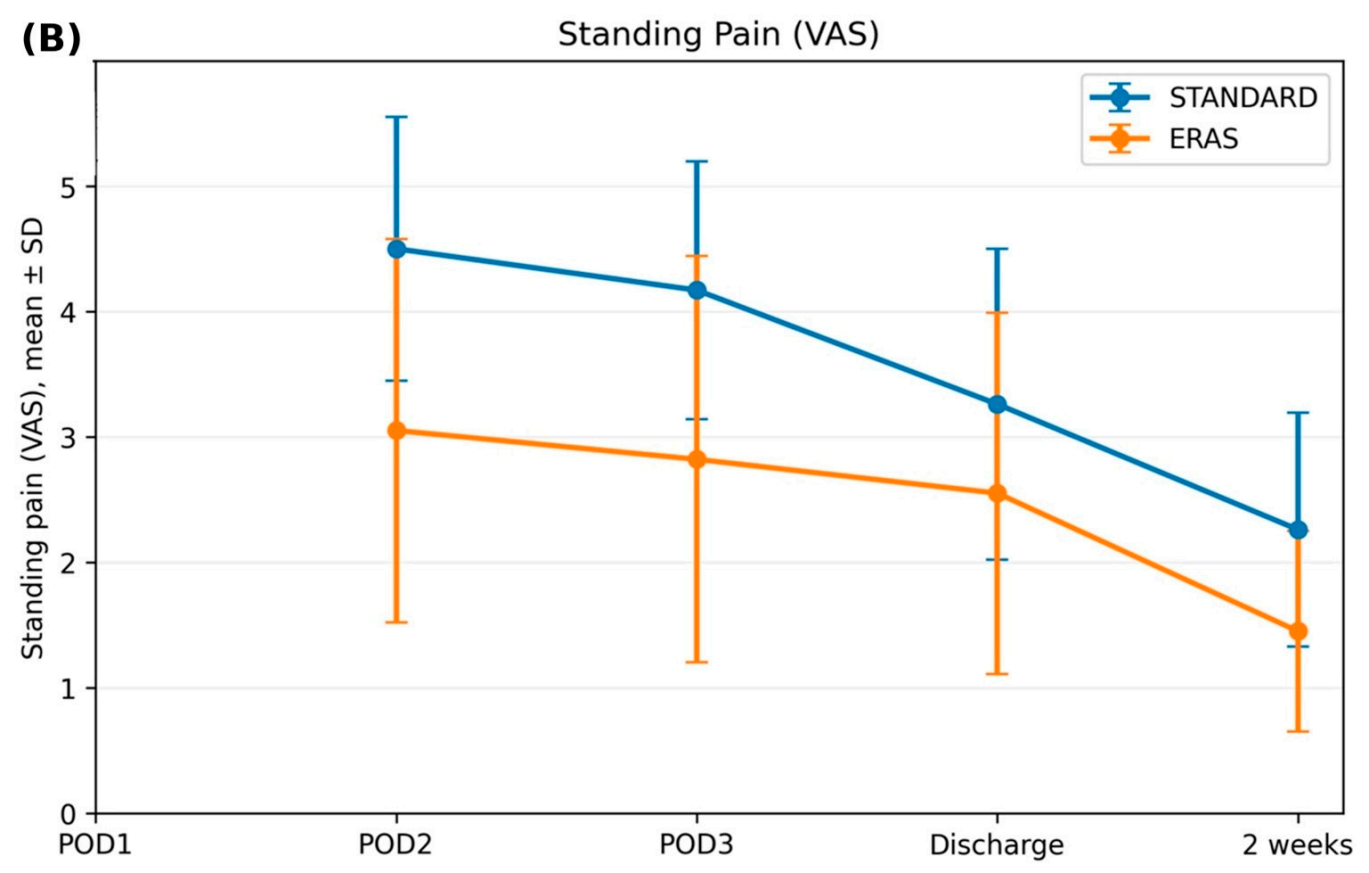
Supine (**A**) and standing (**B**) postoperative pain trajectories.

From POD1 onward, supine pain was comparable between groups, with no statistically significant between-group differences at subsequent timepoints (all *p* > 0.05). Specifically, supine pain was 2.32 ± 0.95 in the standard group versus 2.41 ± 1.50 in the ERAS group on POD1 (*p* = 0.811), 3.00 ± 1.25 vs. 2.41 ± 1.53 on POD2 (*p* = 0.181), and 2.89 ± 1.10 vs. 2.68 ± 1.62 on POD3 (*p* = 0.621). At discharge, mean supine pain was 2.05 ± 0.71 (standard) vs. 2.32 ± 1.36 (ERAS) (*p* = 0.429), and at 2 weeks it was 1.53 ± 0.51 vs. 1.50 ± 0.91 (*p* = 0.909). Pain trajectories are illustrated in Figure 2.

#### 3.2.2. Standing Pain

Standing pain was analyzed via available-case analysis at each timepoint. On POD1, standing pain was not assessable in the standard cohort (0/34), whereas values were available in 22/35 ERAS patients; therefore, no between-group statistical comparison was performed at POD1. From POD2 onward, standing pain was consistently lower in the ERAS group among assessable patients. On POD2, standing pain was 4.50 ± 1.05 in the standard group (n = 6) vs. 3.05 ± 1.53 in the ERAS group (n = 22) (*p* = 0.020). On POD3, it was 4.17 ± 1.03 (standard; n = 12) vs. 2.82 ± 1.62 (ERAS; n = 22) (*p* = 0.006). At discharge, standing pain was numerically lower in ERAS (2.55 ± 1.44) than in standard care (3.26 ± 1.24), but did not reach statistical significance (*p* = 0.094). At 2-week follow-up, standing pain was significantly lower in ERAS (1.45 ± 0.80) versus standard care (2.26 ± 0.93) (*p* = 0.006). Detailed standing pain results are summarized in Table 3.

Standing pain data were dependent on successful completion of an upright mobilization attempt. Mobilization was attempted in both cohorts; however, during the early postoperative phase, patients in the control group were substantially less likely to tolerate standing sufficiently to allow pain assessment. Therefore, early standing pain comparisons should be interpreted as available-case comparisons among patients who successfully completed mobilization, rather than as direct full-cohort comparisons.

### 3.3. Postoperative Nausea (POD0–POD3)

Postoperative nausea was analyzed as a binary endpoint and is reported as n/N (%) in the full cohorts (standard care: n = 34; ERAS: n = 35). Between-group comparisons at each postoperative day were performed using two-sided Fisher’s exact test.

**Figure 3:**
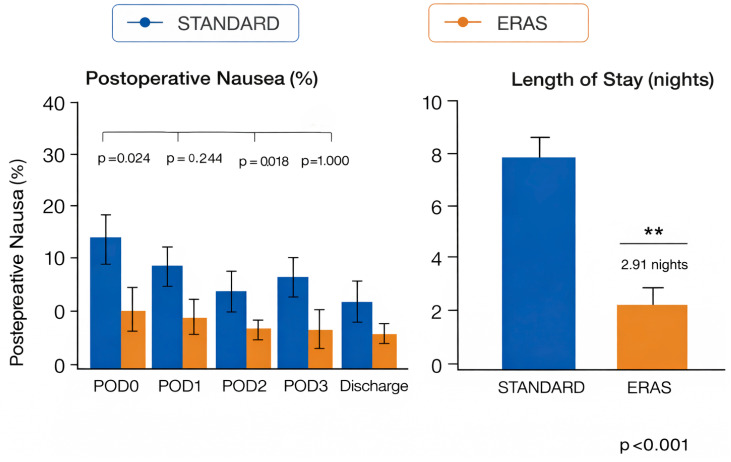
Postoperative nausea (POD0–POD3) and length of hospital stay. ** indicates statistical significance (*p* < 0.01). Blue represents the standard care group, while orange represents the ERAS+ESPB group.

At POD0, nausea occurred in 12/34 (35.3%) patients in the standard-care group versus 4/35 (11.4%) in the ERAS group (*p* = 0.024). At POD1, nausea was observed in 9/34 (26.5%) versus 5/35 (14.3%), respectively (*p* = 0.244). At POD2, rates were 11/34 (32.4%) in standard care and 3/35 (8.6%) in ERAS (*p* = 0.018). At POD3, nausea occurred in 5/34 (14.7%) and 5/35 (14.3%), with no between-group difference (*p* = 1.000). Overall, ERAS was associated with a lower early postoperative nausea burden, with statistically significant differences at POD0 and POD2. Differences at POD1 and POD3 were not significant. Postoperative nausea rates are detailed in Table 4 and graphically represented in Figure 3.

### 3.4. Length of Stay

Length of stay was significantly shorter in the ERAS group than in the standard-care group (5.41 ± 1.10 vs. 8.32 ± 2.06 nights; *p* < 0.001), corresponding to an average reduction of approximately 2.9 nights. Length-of-stay results are summarized in Table 5 and illustrated in Figure 3.

In complete-case multivariable linear regression analysis adjusting for age, sex, main Cobb angle, estimated blood loss, and operative time, treatment within the ERAS pathway remained independently associated with shorter length of stay (β = −2.87 nights, 95% CI −4.10 to −1.64; *p* < 0.001). None of the other covariates were independently associated with length of stay in the adjusted model.

## 4. Discussion

### 4.1. Main Findings

This comparative analysis in adolescents undergoing posterior spinal fusion for idiopathic scoliosis showed a consistent pattern across early recovery domains. The ERAS pathway was associated with substantially lower immediate postoperative resting pain (POD0), faster recovery of pain during functional loading (standing pain from POD2 onward), lower early postoperative nausea burden (especially at POD0), and a substantially shorter hospital stay. By contrast, resting pain trajectories from POD1 to follow-up were broadly similar between groups, suggesting that the main advantage of ERAS emerged in the immediate perioperative window and in mobilization-related recovery rather than in late resting pain intensity. The distinction between supine and standing pain was intentional. Resting pain reflects baseline postoperative analgesic control, whereas standing pain more closely captures the nociceptive burden associated with mobilization and functional recovery. In the context of ERAS, this distinction is clinically meaningful because the goal is not only to reduce pain intensity at rest, but also to facilitate earlier rehabilitation, ambulation, and discharge readiness [10]. However, standing pain should be interpreted as a functional recovery-related endpoint, because it could only be assessed in patients who were able to tolerate upright mobilization. In this sense, the observed between-group difference reflects not only dynamic pain intensity, but also differential early mobilization success.

From a clinical perspective, these findings are relevant because postoperative management in AIS is not judged solely by static pain scores; it is assessed by how rapidly patients can mobilize, tolerate oral intake, and progress toward safe discharge. The reduction in length of stay, combined with preserved pain control at discharge and at two weeks, supports the interpretation of accelerated recovery, without evidence of downstream analgesic penalty.

These findings are consistent with the AIS-ERAS literature. Recent evidence syntheses, including the revised systematic review, support the association between ERAS implementation and shorter hospitalization with preserved safety endpoints in AIS populations [8]. Earlier meta-analyses and systematic reviews reported similar trends, particularly for LOS reduction and improved perioperative efficiency, while highlighting heterogeneity in protocol composition across centers [7,11]. Prospective pathway studies in AIS have also shown that coordinated multimodal care can substantially shorten hospitalization without worsening short-term outcomes [10,12,13]. The present dataset reinforces this line of evidence and adds specific evidence on functional pain (standing pain) rather than resting pain alone. Importantly, these findings should be interpreted at the level of the perioperative care pathway rather than as evidence of an isolated effect of the erector spinae plane block itself. Indeed, the ERAS protocol evaluated in the present study consisted of a bundled multidisciplinary strategy including multimodal analgesia, structured antiemetic management, early mobilization, and bilateral ESPB; because these elements were implemented simultaneously, the specific contribution of each individual component cannot be determined.

### 4.2. Pain Trajectory: Why Standing Pain Improved More Than Supine Pain

The divergence between dynamic and resting pain trajectories is biologically plausible. Dynamic pain better captures the nociceptive burden during mobilization and physiotherapy, which are central targets of ERAS. The early benefit observed in standing pain likely reflects the combined effects of multimodal analgesia, structured rehabilitation, and the overall perioperative ERAS bundle. Because the pathway components were introduced together, the present study cannot isolate the independent contribution of bilateral erector spinae plane block to this finding.

Pediatric AIS evidence for ESPB has grown: randomized controlled trials have shown improvements in postoperative analgesia and/or opioid-related outcomes, and more recent cohort data suggest reduced opioid exposure when ESPB is integrated into standardized pathways [14,15,16]. Therefore, the observed pattern is biologically and clinically plausible and is broadly consistent with prior literature on multimodal perioperative recovery strategies in AIS, including studies in which ESPB was incorporated within structured analgesic protocols.

### 4.3. Nausea, Antiemetic Strategy, and Early Gastrointestinal Recovery

Postoperative nausea remains highly relevant in AIS fusion surgery and is consistently associated with delayed oral intake, delayed mobilization, and prolonged recovery [17]. The lower early nausea burden in the ERAS pathway is compatible with an opioid-sparing strategy and with structured prophylaxis/treatment protocols. Dimenhydrinate has documented antiemetic efficacy in perioperative settings [18], and its inclusion in a multimodal antiemetic approach is biologically coherent. The addition of chewing gum (containing dimenhydrinate in this protocol) is also conceptually aligned with attempts to facilitate gastrointestinal reactivation through sham feeding mechanisms, although pediatric meta-analytic evidence across surgical fields remains mixed and does not uniformly demonstrate accelerated bowel recovery [19]. The intervention should therefore be interpreted as a pragmatic adjunct within a broader ERAS construct rather than as an isolated determinant of outcome.

### 4.4. Length of Stay as a System-Level Endpoint

The magnitude of LOS reduction is among the most practice-relevant findings. In pediatric spine surgery, LOS is not only a healthcare utilization metric but also a proxy for pathway efficiency, pain control adequacy, mobilization success, and discharge readiness. Historical and contemporary AIS studies have repeatedly demonstrated that structured accelerated pathways can shorten LOS without clear evidence of increased early complications or readmissions [10,12,17]. From an implementation perspective, these data support ERAS as an operational framework associated with measurable gains in clinical throughput. This is particularly important in high-volume scoliosis programs where bed turnover and perioperative resource allocation are critical.

From a translational perspective, these findings support the feasibility of implementing ERAS pathways in high-volume pediatric spine centers without requiring major structural changes, suggesting that protocol standardization may represent a scalable strategy to optimize perioperative efficiency.

### 4.5. Clinical Perspective and Next Research Step

Taken together, the current findings support the clinical feasibility and short-term clinical effectiveness of an AIS-specific ERAS pathway incorporating ESPB, particularly with respect to functional recovery and LOS reduction. The next methodological step should be a prospective, preferably multicenter, protocolized evaluation with predefined core outcomes (dynamic pain, opioid exposure, PONV burden, bowel recovery, readiness-for-discharge criteria, and 30-day safety endpoints), to isolate component effects and strengthen causal inference.

The present study was designed to address early postoperative recovery and therefore does not provide a comprehensive assessment of broader postoperative outcome. Longitudinal safety endpoints such as 30-day complications, emergency department visits, readmission, or reoperation were not systematically collected in the retrospective dataset. Future prospective AIS-ERAS studies should incorporate a wider outcome framework, including short-term safety events, medium-term healthcare utilization, and validated patient-reported outcome measures, to better define the overall clinical value of pathway implementation.

Moreover, future studies should explore patient-reported outcomes, cost-effectiveness, and pathway adherence metrics to better characterize the broader impact of ERAS implementation in pediatric spine surgery.

### 4.6. Limitations

Several limitations should be explicitly acknowledged. First, the retrospective and non-randomized study design introduces the possibility of selection bias, temporal bias, and residual confounding. Second, treatment allocation was protocol-era based (standard pathway before full ERAS implementation), so secular trends, team learning effects, and evolving perioperative culture may have contributed to outcome differences independent of the protocol itself. Third, the sample size, although adequate to detect a large LOS effect, limits precision for some secondary outcomes and for subgroup analyses. Fourth, standing pain could only be assessed in patients able to tolerate upright mobilization at each timepoint. Although mobilization was attempted in both cohorts, successful standing assessment was less frequently achievable in the control group during the early postoperative phase. Thus, this available-case structure is clinically meaningful, but it may also introduce ascertainment asymmetry, because standing pain reflects not only dynamic pain intensity but also the patient’s ability to complete early mobilization. Fifth, the ERAS pathway is a bundled intervention: because ESPB, antiemetic strategy, mobilization targets, and other elements were implemented together, causal attribution to any single component cannot be made. Sixth, follow-up was limited to the early postoperative phase, and no medium- or long-term patient-reported outcomes were available. In addition, the study did not systematically capture broader longitudinal safety endpoints such as 30-day complications, emergency department visits, readmission, or reoperation within 1 year. Therefore, the present findings should not be interpreted as a comprehensive evaluation of long-term safety or overall postoperative outcome after AIS surgery.

## 5. Conclusions

In adolescents undergoing posterior spinal fusion for idiopathic scoliosis, an ERAS-based perioperative pathway incorporating bilateral ESP block was associated with improved early postoperative recovery compared with standard care. The main clinical advantages were lower immediate postoperative pain, better dynamic pain control during mobilization, reduced early nausea burden, and a significantly shorter hospital stay. Resting pain after POD1 showed no relevant between-group differences, suggesting that the main benefit of ERAS lies in functional recovery rather than in late static pain reduction. These findings should be interpreted at the pathway level rather than as evidence attributable to ESPB alone. Future prospective multicenter studies incorporating standardized outcome sets and pathway adherence measures are warranted to strengthen causal inference and define the independent contribution of each component.

## Figures and Tables

**Table 1:** Preoperative and intraoperative variables.

	ERAS (n = 35)	Controls (n = 34)	*p* Value
Age (years)	15.2 ± 3.4	15.2 ± 3.4	0.748
Cobb angle (°)	63.3 ± 20	53.8 ± 13.6	0.198
Blood loss (mL)	447.0 ± 254.4	432.5 ± 205.3	0.955
Operative time (min)	227.4 ± 52.9	240.2 ± 58.4	0.345
Sex			0.465
Female	22 (62.9%)	23 (67.6%)	
Male	13 (37.1%)	11 (35.3%)	
Lenke type			0.353
1	15	14	
2	3	2	
3	9	6	
4	1	2	
5	3	4	
6	4	6	

**Table 2:** Supine pain over time (VAS, mean ± SD).

Timepoint	Standard (n = 34)	ERAS (n = 35)	*p*-Value
POD0	3.20 ± 1.50	1.50 ± 0.55	<0.001
POD1	2.32 ± 0.95	2.41 ± 1.50	0.811
POD2	3.00 ± 1.25	2.41 ± 1.53	0.181
POD3	2.89 ± 1.10	2.68 ± 1.62	0.621
Discharge	2.05 ± 0.71	2.32 ± 1.36	0.429
2-week follow-up	1.53 ± 0.51	1.50 ± 0.91	0.909

**Table 3:** Standing pain over time (VAS, available-case analysis).

Timepoint	Standard (n = 34)	ERAS (n = 35)	*p*-Value
POD1	Not assessable	Assessable in 22/35 patients	N/A
POD2	4.50 ± 1.05 (n = 6)	3.05 ± 1.53 (n = 22)	0.020
POD3	4.17 ± 1.03 (n = 12)	2.82 ± 1.62 (n = 22)	0.006
Discharge	3.26 ± 1.24	2.55 ± 1.44	0.094
2-week follow-up	2.26 ± 0.93	1.45 ± 0.80	0.006

**Table 4:** Postoperative nausea (binary outcome, n/N [%]).

Timepoint	Standard (n = 34)	ERAS (n = 35)	*p*-Value
POD0	12/34 (35.3%)	4/35 (11.4%)	0.024
POD1	9/34 (26.5%)	5/35 (14.3%)	0.244
POD2	11/34 (32.4%)	3/35 (8.6%)	0.018
POD3	5/34 (14.7%)	5/35 (14.3%)	1.000

**Table 5:** Length of stay.

Outcome	Standard (n = 34)	ERAS (n = 35)	*p*-Value
Length of stay (nights), mean ± SD	8.32 ± 2.06	5.41 ± 1.10	<0.001
Mean difference (Standard − ERAS)	-	2.91 nights	-

## Data Availability

The original contributions presented in this study are included in the article. Further inquiries can be directed to the corresponding author.
